# Supplementary material for: Source apportionment of potentially toxic elements in soils using APCS/MLR, PMF and geostatistics in a typical industrial and mining city in Eastern China
Source: PLoS One. 2020 Sep 3;15(9):e0238513. doi: 10.1371/journal.pone.0238513 (PMC7470422; doi:10.1371/journal.pone.0238513)
Supplement: Attachment — Submitted filename: Response-to-Reviewers_cjf.doc [file pone.0238513.s002.doc]

**Original Manuscript ID:** PONE-D-20-05425

**Original Article Title:** "Source apportionment of potentially toxic elements in soils using APCS/MLR, PMF and geostatistics in a typical industrial and mining city in Eastern China"

Dear Editor and Reviewers,

Thank you for allowing a resubmission of our manuscript, with an opportunity to address the reviewers’ comments. We are uploading (a) our point-by-point response to the comments (below) (response to reviewers), (b) a final manuscript with highlights (Revised Manuscript with Track Changes) (c) a clean final manuscript without highlights (Manuscript).

Best regards,

Authors: Cao Jianfei, Li Chunfang, Zhang Lixia, Wu Quanyuan, Lv Jianshu

**Journal Requirements #1, Concern # 1:** PLOS specifies that experiments, statistics, and other analyses are performed to a high technical standard; sample sizes are large enough to produce robust results; and methods are described in sufficient detail to allow another researcher to reproduce the experiment (http://journals.plos.org/plosone/s/criteria-for-publication#loc-3). We feel that your methods section did not include sufficient detail, including the manufacturer of all chemicals and instruments. Additionally, if the protocols used are previously published ones, citations should be given. Please revise this section to include this information.

**Author response:** Thank you for your contribution to this article. In the revised manuscript, we added the details of the experiment, including the manufacturer of all chemicals and instruments.

“

A total of 138 soil samples were collected in the summer of 2017 based on the grid layout sample point method. Sample sites were selected according to a sampling density of less than 2 km based on Landsat images, and each sample consisted of a mixture of five subsamples collected from five spots across an area of approximately 30 m2. If the designed site was unavailable for sampling (such as if it contained a road or building), an alternative location was selected as close to the original as possible to find natural soils. All subsamples were collected at a depth of 0-20 cm using a stainless-steel shovel. After natural air drying and debris removal, the soil samples were sieved to 0.84 mm and ground to a fine powder with a particle size of less than 0.25 mm. One kilogram of the soil samples was transported to a laboratory for potentially toxic element content analysis. The geographical locations of the sampling points were recorded by a GPS receiver, as shown in Fig 1.

In the laboratory, the soil was pretreated by air drying and was ground to a fine powder with a particle size of less than 0.149 mm. Soil pH values were measured by a pH meter in a 1:2.5 soil–water suspension, and Soil organic matter (SOM) contents were analyzed using oil bath-K2CrO_7_ titration. HSO_4_-HNO_3_-HF was used to digest the soil for analyzing the As, Cd, Cr, Cu, Hg, Ni, Pb and Zn contents. The Cr, Cu, Ni, Pb and Zn contents were analyzed using a flame atomic absorption spectrophotometer (240 AA Agilent, USA), Cd contents were determined using a graphite furnace atomic absorption spectrophotometer (AA-7000 Shimadzu, Japan), and As and Hg concentrations were determined with an atomic fluorescence spectrophotometer (AFS230E Haiguang Analytical Instrument Co., Beijing, China). For details on the measurements, please refer to the related literature [24, 31]. The recovery rate and standard reference material were examined under strict monitoring, and the chemical analysis process followed the standard for geochemical evaluation of land quality (DZ/T0295—2016) in China. The limit of recovery was 100±10%.

”

[24] Lv JS, Zhang ZL, Liu Y, Dai J, Wang X, Wang M. Sources identification and hazardous risk delineation of potentially toxic elements contamination in Rizhao City. Acta Geogr Sin. 2012; 67(7):971-84. doi:10.11821/xb201207010.

[31] Lu RK. Analysis Method of Soil and Agricultural Chemistry. Beijing: China Agricultural Science and Technology Press; 2000.

**Journal Requirements #1, Concern # 2:** Please ensure that you refer to Figure 7 in your text as, if accepted, production will need this reference to link the reader to the figure.

**Author response:** We mistakenly wrote Fig. 6 as Fig. 7. We have revised it in the revised manuscript.

**Journal Requirements #1, Concern # 3:** Please upload a copy of Figure 6, to which you refer in your text. If the figure is no longer to be included as part of the submission please remove all reference to it within the text.

**Author response:** We mistakenly wrote Fig. 6 as Fig. 7. We have revised it in the revised manuscript.

**Journal Requirements #1, Concern # 4:** We note that [Figure(s) 1-7] in your submission contain [map/satellite] images which may be copyrighted. All PLOS content is published under the Creative Commons Attribution License (CC BY 4.0), which means that the manuscript, images, and Supporting Information files will be freely available online, and any third party is permitted to access, download, copy, distribute, and use these materials in any way, even commercially, with proper attribution. For these reasons, we cannot publish previously copyrighted maps or satellite images created using proprietary data, such as Google software (Google Maps, Street View, and Earth). For more information, see our copyright guidelines: http://journals.plos.org/plosone/s/licenses-and-copyright.

**Author response:** The China map (Fig 1) was generated using free, open access data sources from the National Geomatics Center of China. The land used map (Fig 2) was generated by interpreting free Landsat images. The parent materials map (Fig 3) was generated using free, open access data sources from the Geological survey development research center of China. Therefore, the images in our submission complies with the CC BY 4.0 license, and have added a note in the figure caption text.

**Editor#1, Concern # 1:** Arsenic was analyzed, but As is not a heavy metal. In my opinion, "heavy metal" should be change by "Potentially Toxic Elements". Besides, it should be interesting to explain why these elements were analyzed (introduction or mat and methods). For example, Ba or Mn are urban contaminants due to their origin from industrial and traffic sources (Ba). Even Fe is an interesting tracer for geochemical assessment. I understand that As and Hg are related to industrial activities. Maybe it should be better to try to improve why these elements were selected.

**Author response:** Thanks for your comments. We agree with you and have changed the “heavy metal” to “Potentially Toxic Elements”. Arsenic (As) has the property of gold and is easily oxidized in moist air. It mainly exists in nature in the form of sulfide minerals (such As_4_S_4_, As_2_S_3_, etc.). The Cd, Cr, As, Hg, Pb, Cu, Zn and Ni have been listed as priority control pollutants by the United Environmental Protection Agency [27]. Currently, the potentially toxic elements of most concern arsenic (As), cadmium (Cd), chromium (Cr), copper (Cu), mercury (Hg), nickel (Ni), lead (Pb), and zinc (Zn) in city soils in east china, such as the work of Boshan [1], the work of Nanjing [2], the work of typical sewage irrigation area of Shandong Province[3]. Ba, Co, Mn and other heavy metal elements will be studied in our next work.

[1] Lv JS, Liu Y. An integrated approach to identify quantitative sources and hazardous areas of heavy metals in soils. Science of the Total Environment. 2019; 646:19-28.

[2] Hu WY, Wang HF, Dong LR, Huang B, Borggaard OK, Hans HCB, He Y, Holmbe PE. Source identification of heavy metals in peri-urban agricultural soils of southeast China: an integrated approach. Environmental Pollution. 2018; 237:650-661.

[3] Wang F, Li CF, Wang JN, Cao WT, Wu QY. Concentration estimation of heavy metal in soils from typical sewage irrigation area of Shandong Province, China using reflectance spectroscopy [J]. Environmental Science & Pollution Research, 2017, 24(3):16883-16892.

**The main modifications were shown as follows (Lines 105):**

“Potentially toxic elements of As, Cd, Cr, Cu, Hg, Ni, Pb and Zn in 138 surface soil samples were collected [27].”

[27] United States Environmental Protection Agency. Code of Federal Regulations: Priority Pollutants List. Washington: The Agency; c2018 [cited 2018 Dec 11]. Available from: https://www.gpo.gov/fdsys/pkg/CFR-2014-title40-vol29/xml/CFR-2014-title40-vol29-part423-appA.xml.

**Editor#1, Concern # 2:** Information about soil origin is missing. I saw that are several samples and it is impossible to write in a table. However, it should be interesting to try to write a short sentence about the origin of the sample. E.g. XX from parks and gardens, XX near to roads, XX industrial areas, XX agricultural areas. See for example https://doi.org/10.1007/s11368-017-1750-0 https://doi.org/10.1007/s11368-015-1304-2 https://doi.org/10.1007/s12665-019-8762-6.

**Author response:** Thanks for your advice. A total of 138 soil samples were collected in the summer of 2017 based on the grid layout sample point method. This sampling method aims to provide enough sampling points to provide reliable basic data for accurate source analysis of potentially toxic elements in soil [1-4]. Each soil sampling site was first classified based on land use types including 33 for industrial and mining use, 34 for grain crop use, 24 for orchard use, 12 for vegetable use, 22 for residential use, and 13 near to roads.

[1] Lv JS, Liu Y. An integrated approach to identify quantitative sources and hazardous areas of heavy metals in soils. Science of the Total Environment. 2019; 646:19-28.

[2] Yang Liu, Jianshu Lv, Bing Zhang, Jun Bi, Spatial multi-scale variability of soil nutrients in relation to environmental factors in a typical agricultural region, Eastern China, Science of The Total Environment, 2013 (450–451), 108-119.

[3] Qu M , Wang Y , Huang B , et al. Source apportionment of soil heavy metals using robust absolute principal component scores-robust geographically weighted regression (RAPCS-RGWR) receptor model. Science of the Total Environment, 2018, 626:203-210.

[4] Qu M , Wang Y , Huang B , et al. Source apportionment of soil heavy metals using robust absolute principal component scores-robust geographically weighted regression (RAPCS-RGWR) receptor model. Science of the Total Environment, 2018, 626:203-210.

**The main modifications were shown as follows (Lines 149-153):**

“

Sample sites were selected according to a sampling density of less than 2 km based on Landsat images, and each sample consisted of a mixture of five subsamples collected from five spots across an area of approximately 30 m^2^. Each soil sampling site was first classified based on land use types including 33 for industrial and mining use, 34 for grain crop use, 24 for orchard use, 12 for vegetable use, 22 for residential use, and 13 near to roads. If the designed site was unavailable for sampling (such as if it contained a road or building), an alternative location was selected as close to the original as possible to find natural soils.

”

**Editor#1, Concern # 3:** The Source apportionment method is very interesting and gives an alternative approach to typical papers on urban contamination. However, in my opinion, it should be well explained the APCS/MLR and PMF. A short explanation about why this method is more suitable than typical approaches (e.g. advantages) and a short explanation for readers to try to replicate for their works. In a previous work https://doi.org/10.1016/j.envpol.2018.09.147 the authors give more details, and it could be interesting also for this paper. If authors do not want to add into the manuscript, it could be interesting to add as supplementary data like this paper https://doi.org/10.1007/s00244-018-0572-4

**Author response:** Thanks for your advice. In the revision manuscript, we introduce the advantages of the proposed source apportionment method in more detail under “Introduction” section. It included the quantitative analysis advantage of the receptor model over the statistical method (Lines 56-77), the robustness advantage of the combination of the multi-receptor model (Lines 76-82), and the advantage of the geostatistical method considering the spatial relationship of the sampling points (Lines 83-103).

**Editor#1, Concern # 4:** Why was selected Ordinary kriging and not Inverse distance weighting (IDW) or SPLINE?

**Author response:** Thanks for your kindly suggestion. In the revision manuscript, we have added an explanation for the choice of Ordinary Kriging under “Introduction” section.

**The main modifications were shown as follows (Lines 81-90):**

“The spatial correlations between sampling points contain important information for interpret the potential soil potentially toxic element pollution source. There are two main groups of interpolation techniques: deterministic (polynomial, inverse distance weighted, and radial) and geostatistical (ordinary kriging, simple kriging, universal kriging, probability kriging, indicator kriging and disjunctive kriging) [21]. Because the geostatistical method coupled with the variography and kriging methods could quantify the spatial autocorrelation among measured points and account for the spatial configuration of the sample points around the prediction location, they have been widely used to provide insights into the spatial correlations of soil properties.”

[21] Johnston K, Hoef JMV, Krivoruchko K, Lucas K, Using ArcGISTM Geostatistical Analyst. New York: Environmental Systems Research Institute; 2001.

**Editor#1, Concern # 5:** Line 203 "A higher SOM in some soils could be related to high application rates of organic fertilizers". First, this type of sentences should be written as discussion and soil properties are missing in the discussion section. In any case, the manuscript is missing some information about soil origin. I think that these higher values can be related to parks and gardens.

Besides, it should be interesting to try to see if contents of Cd, Cr or Cu are related to this SOM contents (Line 328). Sometimes are also related to garden-tending processes (protectors or wooden fences treated with chromate) as the most probable origin. Besides, P and N contents can help to explain well this relation between SOM and organic fertilizers <https://doi.org/10.1007/s11368-015-1304-2>

**Author response:** We agree with your opinion. However, the proposed source apportionment judge potential pollution sources based on multiple information, including robust source factors, the spatial variant structure of factor variables and the spatial distribution characteristics of the factors and eight potentially toxic elements. SOM was not a required data in the proposed method, just for reference. Therefore, we did not describe SOM too much in this paper.

**Editor#1, Concern # 6:** why other soils properties (e.g. P, N, soil texture, etc.) were not measured? In addition, how was measured pH and OM? (Calcination, Walkey black, etc)

**Author response:** The proposed source apportionment judge potential pollution sources based on multiple information, including robust source factors, the spatial variant structure of factor variables and the spatial distribution characteristics of the factors and eight potentially toxic elements. Soils properties (e.g. P, N, soil texture, etc.) were not a required data in the proposed method. Therefore, other soils properties (e.g. P, N, soil texture, etc.) were not measured.

In addition, we have supplemented the soil pH and OM measurement description in the revised manuscript **(Lines 161-163).**

“Soil pH values were measured by a pH meter in a 1:2.5 soil–water suspension, and Soil organic matter (SOM) contents were analyzed using oil bath-K_2_CrO_7_ titration.”

**Editor#1, Concern # 7:** I suggest a short paragraph with a comparison of studied soils with soils from other Chinese cities.

**Author response:** Thanks for your kindly suggestion. In the eastern areas of China, intensive human activities have led to the enrichment of soil potentially toxic elements in soils, threatening food safety and human health. Longkou has a relatively complete industrial and mining system and was selected as a typical area, eastern China. Overall, the average potentially toxic element contents (As, Cd, Cr, Cu, Hg, Ni, Pb and Zn) in all samples were below level II of the Environmental Quality Standard for Soils (EQSS) of China [36] but exceeded the corresponding background values [35]. Compared with the surrounding cities with developed industry and mining, such as Rizhao [24], Guangrao [25] and Ju country [26], it was found that the average value of potentially toxic elements in soils had the above similar characteristics, and Cd and Hg were also considered to be the most risky.

**The main modifications were shown as follows (Lines 246-250).**

“

Overall, the average potentially toxic element contents in all samples were below level II of the Environmental Quality Standard for Soils (EQSS) of China [Error: Reference source not found] but exceeded the corresponding background values [Error: Reference source not found]. In particular, Cd, Cu and Hg were 1.81, 1.80 and 1.63 times higher than the background values, respectively, suggesting that the topsoil, which is affected by human activities, was enriched by these potentially toxic elements. Compared with the surrounding cities with developed industry and mining, such as Rizhao [24], Guangrao [25] and Ju country [26], it was found that the average value of potentially toxic elements in soils had the above similar characteristics as in Longkou, and Cd and Hg were also considered to be the most risky.

”

[24] Lv JS, Zhang ZL, Liu Y, Dai J, Wang X, Wang M. Sources identification and hazardous risk delineation of heavy metals contamination in Rizhao City. Acta Geogr Sin. 2012; 67(7):971-84. doi:10.11821/xb201207010.

[25] Lv JS, Liu Y, Zhang ZL, Dai JR, Dai B, Zhu YC. Identifying the origins and spatial distributions of heavy metals in soils of Ju country (Eastern China) using multivariate and geostatistical approach. J Soils Sediments. 2015; 15(1):163-78. doi:10.1007/s11368-014-0937-x.

[26] Lv JS. Multivariate receptor models and robust geostatistics to estimate source apportionment of heavy metals in soils. Environ Pollut. 2019;244:72-83. doi:10.1016/j.envpol.2018.09.147.

**Editor#1, Concern # 8:** I think that references are not according to the journal guidelines. E.g. Journal names should be abbreviated [https://journals.plos.org/plosone/s/submission-guidelines#loc-references](https://journals.plos.org/plosone/s/submission-guidelines#loc-references)

**Author response:** Thanks for your comments. In the revised manuscript, the format has been adjusted to make it consistent with the journal guidelines.

**Editor#1, Concern # 9:** Line 38. Typo error. Line 181. "eight" instead "8". Please, try to write the full name for numbers 1-10 when it's possible.

**Author response:** We have revised those sentences.

**Editor#1, Concern # 10:** L126. Why 0.074 nm mesh?

**Author response:** We have revised the sentences. The main modifications were shown as follows **(Lines 160-161):**

“In the laboratory, the soil was pretreated by air drying and was ground to a fine powder with a particle size of less than 0.149 mm.”

**Editor#1, Concern # 11:** Line 135. Which means the reference #23? Is it related to the methodological method?

**Author response:** Thanks for your comments. The chemical analysis were measured according to the related the reference #23. In the revised manuscript, we put it in appropriate citation position. **The main modifications were shown as follows (Line 163-170)**:

“

The Cr, Cu, Ni, Pb and Zn contents were analyzed using a flame atomic absorption spectrophotometer (240 AA Agilent, USA), Cd contents were determined using a graphite furnace atomic absorption spectrophotometer (AA-7000 Shimadzu, Japan), and As and Hg concentrations were determined with an atomic fluorescence spectrophotometer (AFS230E Haiguang Analytical Instrument Co., Beijing, China). For details on the measurements, please refer to the related literature [24, 31].

”

[24] Lv JS, Zhang ZL, Liu Y, Dai J, Wang X, Wang M. Sources identification and hazardous risk delineation of heavy metals contamination in Rizhao City. Acta Geogr Sin. 2012; 67(7):971-84. doi:10.11821/xb201207010.

[31] Lu RK. Analysis Method of Soil and Agricultural Chemistry. Beijing: China Agricultural Science and Technology Press; 2000.

**Editor#1, Concern # 12:** Line 200. Please, also give for the pH values the max and min such as SOM contents.

**Author response:** We have revised the sentences.

**Editor#1, Concern # 13:** Table 2 it should be after Line 225

**Author response:** We have revised it.

**Reviewer#1, Concern # 1:** The authors should rewrite the abstract as: First explain the background of your work, objectives, then methods employed and main results of this study and finally what are the recommendations of this work to the society/enterprises/environmentalists.

**Author response:** Thank for your comment. We rewrote the abstract.

**The main modifications were shown as follows (Lines 39-42):**

**Background:** Source apportionment of potentially toxic elements in soils is a critical step for devising soil sustainable management strategies. However, misjudgment or imprecision can occur when traditional statistical methods are applied to identify and apportion the sources.

**Objective:** The main objective of the study was to develop a robust approach composed of the absolute principal component score/multiple linear regression (APCS/MLR) receptor model, positive matrix factorization (PMF) receptor model and geostatistics to identify and apportion sources of soil potentially toxic elements in typical industrial and mining city, eastern China.

**Methods:** APCS/MLR and PMF were applied to provide robust factors with contribution rates. The geostatistics coupled with the variography and kriging methods was used to present factors derived from these two receptor models.

**Main results：**The results indicated that mean concentrations of As, Cd, Cr, Cu, Hg, Ni, Pb and Zn exceeded the local background levels. Based on multivariate receptor models and geostatistics, we determined four sources of eight potentially toxic elements including natural source (parent material), agricultural practices, pollutant emissions (industrial, mining and traffic) and the atmospheric deposition of coal combustion, which accounted for 68%, 12%, 12% and 9% of the observed potentially toxic element concentrations, respectively.

**The recommendations**：This study provides a reliable and robust approach for potentially toxic elements source apportionment in this particular industrial and mining city with a clear potential for future application in other regions.

**Reviewer#1, Concern # 2:** The introduction section is very weak, authors should revise this section by following these articles:

Ecological Risk Assessment and Source Apportionment of heavy metal contamination in Agricultural Soils of North-eastern Iran.

Temporal distribution, source apportionment and pollution assessment of metals in the sediments of river Beas, India.

Assessment of heavy metal pollution in three different Indian water bodies by combination of multivariate analysis and water pollution indices.

Assessment of soil properties from catchment areas of Ravi and Beas Rivers: A review.

Pollution assessment of heavy metals in soils of India and ecological risk assessment: A State-of-the-Art.

Pollution assessment and spatial distribution of roadside agricultural soils: A case study from India.

Spatial distribution and potential ecological risk assessment of heavy metals in agricultural soils of Northeastern, Iran

Assessment of pollution in roadside soils by using multivariate statistical techniques and contamination indices.

Global evaluation of heavy metal content in surface water bodies: A meta-analysis using heavy metal pollution indices and multivariate statistical analyses.

Appraisal of metallic pollution and ecological risks in agricultural soils of Alborz province, Iran.

Ecological and human health risks appraisal of metals in agricultural soils: a review.

Before objective, author should explain the rational of this study.

**Author response:** Thanks for your kindly suggestion. We updated the manuscript by adding the rational of this study and related references in Section Introduction and highlighting these sentences (Line 55-103). The main modifications included explaining the advantages of combing of APCS/MLR and PMF，the role of Kring, and the typicality of the study area.

[10] Keshavarzi A, Kumar V. Ecological risk assessment and source apportionment of heavy metal contamination in agricultural soils of Northeastern Iran. Int J Environ Heal R, 2018; 29(6):1-17.

[11] Hou DY, O'Connor D, Nathanail P, Tian L, Ma Y. Integrated GIS and multivariate statistical analysis for regional scale assessment of heavy metal soil contamination: A critical review. Environ Pollut. 2017; 231:1188-200.

[12] Kumar V, Sharma A, Minakshi, B, Renu, T, Ashwani K．Temporal distribution, source apportionment, and pollution assessment of metals in the sediments of Beas river, India．Hum Ecol Risk Assess. 2018, 24(7-8):2162-2181.

**Reviewer#1, Concern # 3:** Line 93, Materials and methods should be rewritten as material and methods.

**Author response:** We have revised it.

**Reviewer#1, Concern # 4:** In study area, authors have not added any reference, from where this information was taken. Author should add references in this section. Why this area is important for this work. Explain it also.

**Author response:** Thanks for your kindly suggestion. In the eastern coastal areas of China, intensive human activities have led to the enrichment of soil heavy metals in soils, threatening food safety and human health. We develop a robust approach composed of the absolute principal component score/multiple linear regression (APCS/MLR) receptor model, positive matrix factorization (PMF) receptor model and geostatistics to identify and apportion sources of soil potentially toxic elements in typical industrial and mining city, eastern China, Longkou has a relatively complete industrial and mining system compared with other eastern cities and was selected as a typical area. In the revised manuscript, we added a description of why longkou city was chosen.

**The main modifications were shown as follows (Lines 114-122):**

“Longkou, a typical industrial and mining city in eastern China [28]. The research was conducted in the northern plain of Longkou City (37°34'35"N - 37°44´49"N, 120°13´4"E - 120°40´47"E), which covers an area of 500 km2 (Fig 1). The study areas are priority areas in which industries have rapidly developed under the support of state policies. The multitude of emission sources have made this a typical area for verifying source apportionment models [29]. The area is characterized by mineral resource exploitation, including coal, gold mine and lead zinc mining, and the abundant natural resources have promoted the development of preliminary industrial enterprises, such as iron-making plants, paper mills and electroplating factories [30].”

[28] Liu S, Wu QY, Cao XJ, Wang JN, Zhang LL, Cai DQ, Zhou LY, Liu N. Pollution Assessment and Spatial Distribution Characteristics of Heavy Metals in Soils of Coal Mining Area in Longkou City. Environ Sci, 2016, 37(1): 270-279.

[29] Li CF, Wang F, Cao WT, Pan J, Lv JS, Wu QY. Source analysis, spatial distribution and pollution assessment of heavy metals in sewage irrigation area farmland soils of Longkou City. Environ Sci. 2017; 38(3):1018-1027.

[30] Cao JF, Li CF, Wu QY, Qiao JM. Improved Mapping of Soil Heavy Metals Using a Vis-NIR Spectroscopy Index in an Agricultural Area of Eastern China. IEEE Access. 2020; 8:42584-42594.

**Reviewer#1, Concern # 5:** Table 1, authors have calculated C.V., S.D. and skewness, please discuss the following in the results and support your results by adding appropriate references.

**Author response:** Thanks for your comment. We updated the manuscript by adding a discussion for supporting the results.

**The main modifications were shown as follows (Lines 257-262):**

“

The coefficient of variation (C.V.) is a dimensionless expression of the standard deviation and can better reflect fluctuations in potentially toxic element contents [38]. The highest C.V. was found for Pb followed by Cr and Cd, which indicates high variations of these metals in the soil, and exhibited the following order: Cd>Cu>Hg>Pb>Ni>As>Zn>Cr. The skewness of the studied potentially toxic elements exhibited the following order: Cd>Pb>Cu>Hg>Zn>Ni>As>Cr. Overall, Cd, Pb, Cu, Hg and Zn were were found to be highter than one which indicates right handed skewness [39]. It suggest that these soil metals may be affected by human factors [38].

”

[38] Kumar V, Sharma A, Kaur P, Singh Sidhu GP, Bali AS, Bhardwaj R, et al. Pollution assessment of heavy metals in soils of India and ecological risk assessment: A state-of-the-art. Chemosphere. 2019; 216:449-462.

[39] Beaver MB, Beaver JR, Mendenhall W. Introduction to probability and statistics. New Delhi: Cengage Learning. 2012.

**Reviewer#1, Concern # 6:** In Table 2, the communality of the factor loadings missing, authors should add this in the Table 2.

**Author response:** We have revised it. The last row (Accumulated Variance Contribution Rate/%) of the Table 2 corresponds to this factor the communality of the factor loadings.

**Table 2 Factors loadings of potentially toxic elements in soils**.

|  | F1 | F2 | F3 | F4 |
| --- | --- | --- | --- | --- |
| Cr | 0.923 | -0.075 | 0.168 | -0.004 |
| Cu | -0.103 | 0.894 | 0.06 | -0.098 |
| Ni | 0.916 | -0.055 | 0.036 | -0.156 |
| Pb | 0.137 | -0.005 | 0.925 | 0.139 |
| Zn | 0.137 | 0.772 | 0.002 | 0.272 |
| Cd | 0.267 | 0.123 | 0.683 | -0.483 |
| As | 0.67 | 0.288 | 0.269 | 0.188 |
| Hg | 0.005 | 0.13 | 0.019 | 0.924 |
| Variance contribution rate/% | 27.145 | 18.938 | 17.635 | 15.876 |
| Accumulated Variance Contribution Rate/% | 27.145 | 46.083 | 63.718 | 79.594 |

**Reviewer#1, Concern # 7:** Authors should add paragraph about the importance of this work to the society at the end of conclusion section.

**Author response:** Thanks for your advice. In the revised manuscript, we added paragraph about the importance of this work to the society at the end of conclusion section.

**The main modifications were shown as follows (Lines 454-456, Lines 474-475):**

“

This study provides a reliable and robust approach for potentially toxic elements source apportionment in this particular industrial and mining city with a clear potential.

”

“

In addition, this research idea can be applied to other research areas.

”

**Reviewer#1, Concern # 8:** Also improve the language of this manuscript.

**Author response:** We have improved the quality of language of this manuscript by a 3rd party service for language polishing (AJE). In the revised manuscript, we carefully checked and improve the language.

**Reviewer#2, Concern # 1:** Line 38 Print error.

**Author response:** We have revised it.

**Reviewer#2, Concern # 2:** Line 200 How did you determine pH and soil organic matter?

**Author response:** We have supplemented the soil pH and OM measurement description in the revised manuscript (Lines 161-163).

“Soil pH values were measured by a pH meter in a 1:2.5 soil–water suspension, and Soil organic matter (SOM) contents were analyzed using oil bath-K_2_CrO_7_ titration.”

**Reviewer#2, Concern # 3:** Line 204 Do not repeat data from table in text.

**Author response:** We have revised it. In the revised manuscript, we have removed the unnecessary data descriptions.

**Reviewer#2, Concern # 4:** Table 1 should have median, percentile 25 and percentile 75.

**Author response:** In the revised manuscript, we have added the data of median, percentile 25 and percentile 75.

**Table 1 Descriptive statistics of potentially toxic elements in the study area (n=138, units in mg kg^-1^**).

| Species | Min | Max | median | percentile  25 | percentile  75 | Mean | S.D. | C.V.(%) | Skewness | Background | I_geo_ | EQSS |
| --- | --- | --- | --- | --- | --- | --- | --- | --- | --- | --- | --- | --- |
| As | 3.63 | 10.26 | 7.98 | 6.73 | 8.97 | 7.96 | 1.47 | 18.51 | 0.34 | 6.30 | -0.25 | 30 |
| Cd | 0.049 | 0.42 | 0.23 | 0.13 | 0.37 | 0.20 | 0.11 | 56.00 | 9.91 | 0.11 | 0.28 | 0.30 |
| Cr | 46.7 | 96.83 | 61.08 | 50.10 | 64.8 | 61.10 | 10.58 | 17.32 | 0.23 | 56.20 | -0.46 | 200 |
| Cu | 20.34 | 50.86 | 35.35 | 21.73 | 43.78 | 35.30 | 16.60 | 47.02 | 6.61 | 19.60 | 0.26 | 100 |
| Hg | 0.025 | 0.064 | 0.048 | 0.038 | 0.057 | 0.049 | 0.02 | 42.86 | 2.55 | 0.03 | 0.12 | 0.50 |
| Ni | 19.18 | 47.6 | 26.62 | 22.91 | 31.79 | 26.59 | 5.26 | 19.78 | 0.38 | 23.50 | -0.41 | 50 |
| Pb | 15.75 | 63.92 | 35.13 | 28.90 | 50.36 | 35.08 | 13.37 | 38.11 | 8.20 | 25.40 | -0.12 | 300 |
| Zn | 53.99 | 104.32 | 77.85 | 60.11 | 91.57 | 77.89 | 14.05 | 18.04 | 1.02 | 56.10 | -0.11 | 250 |
| PH | 6.26 | 7.88 | 7.00 | 6.68 | 7.38 | 7.00 | 0.35 | 5.00 | 0.37 | - | - | - |
| SOM  （g kg^-1^） | 5.46 | 42.22 | 14.99 | 8.86. | 18.25 | 14.94 | 5.04 | 33.73 | 1.44 | 13.00 | - | - |

**Reviewer#2, Concern # 5:** Table 4. The Gaussian model should not test as it yields implausible results from its use (Oliver and Webster, 2014).

Oliver, M.A., Webster, R., 2014. A tutorial guide to geostatistics: computing and modelling variograms and kriging. Catena 113, 56–69.

**Author response:** Thanks for your comment. We agree with you that Gaussian model is at the limit of acceptability for a random process and can lead to bizarre predictions [1-2]. We eliminated the gaussian model from the model candidate library and calculated again to obtain the best model. Although it didn't change the final results of source apportionment, which made our research more reliable,

[1] Chilès, J.-P., Delfiner, P., 2012. Geostatistics: Modeling Spatial Uncertainty, second edition.

John Wiley & Sons, New York

[2] Oliver, M.A., Webster, R., 2014. A tutorial guide to geostatistics: computing and modelling variograms and kriging. Catena 113, 56–69.

In the revised manuscript, we update the results of geostatistical methods. **The main modifications were shown as follows (Line 304):**

Table 4 Optimal variation function model of the source factors and soil potentially toxic elements

|  | Model | Nugget（C_0_） | Sill  （C_0_+C） | Proportion  C_0_/(C_0_+C) | Range  (A)/m | RSS | R^2^ |
| --- | --- | --- | --- | --- | --- | --- | --- |
| Lg APCS1 | Exponential | 0.008 | 0.039 | 0.210 | 6790 | 0.024 | 0.620 |
| Lg APCS2 | Spherical | 0.051 | 0.105 | 0.486 | 7872 | 0.037 | 0.763 |
| Lg APCS3 | Exponential | 0.068 | 0.108 | 0.627 | 6930 | 0.071 | 0.728 |
| Lg APCS4 | Exponential | 0.048 | 0.106 | 0.453 | 5115 | 0.020 | 0.587 |
| Lg PMF-F1 | Exponential | 0.012 | 0.005 | 0.240 | 4643 | 0.020 | 0.568 |
| Lg PMF-F2 | Spherical | 0.042 | 0.074 | 0.569 | 9492 | 0.011 | 0.812 |
| Lg PMF-F3 | Exponential | 0.055 | 0.076 | 0.720 | 7800 | 0.042 | 0.617 |
| Lg PMF-F4 | Exponential | 0.041 | 0.082 | 0.500 | 5974 | 0.002 | 0.783 |
| Lg As | Exponential | 0.022 | 0.106 | 0.207 | 8768 | 0.013 | 0.728 |
| Lg Cd | Spherical | 0.073 | 0.105 | 0.700 | 7852 | 0.002 | 0.780 |
| Lg Cu | Spherical | 0.045 | 0.069 | 0.652 | 9796 | 0.011 | 0.642 |
| Lg Cr | Exponential | 0.033 | 0.282 | 0.117 | 5947 | 0.012 | 0.590 |
| Lg Hg | Exponential | 0.022 | 0.037 | 0.595 | 7567 | 0.056 | 0.683 |
| Lg Ni | Spherical | 0.011 | 0.085 | 0.129 | 4967 | 0.002 | 0.783 |
| Lg Pb | Exponential | 0.065 | 0.131 | 0.495 | 7200 | 0.042 | 0.647 |
| Lg Zn | Spherical | 0.032 | 0.098 | 0.327 | 6860 | 0.003 | 0.576 |

Fig. 5 Kriged interpolation of the APCS and PMF factors. (The interpolated map plotted with the optimal ordinary kriged interpolation model)

Fig. 6 Kriged interpolation of the concentration of potentially toxic elements. (The interpolated map plotted with the optimal ordinary kriged interpolation model)
