# Supplementary material for: Source apportionment of potentially toxic elements in soils using APCS/MLR, PMF and geostatistics in a typical industrial and mining city in Eastern China
Source: PLoS One. 2020 Sep 3;15(9):e0238513. doi: 10.1371/journal.pone.0238513 (PMC7470422; doi:10.1371/journal.pone.0238513)
Supplement: Attachment — Submitted filename: Response-to-Reviewers_cjf 0717.doc [file pone.0238513.s003.doc]

**Original Manuscript ID:** PONE-D-20-05425R1

**Original Article Title:** "Source apportionment of potentially toxic elements in soils using APCS/MLR, PMF and geostatistics in a typical industrial and mining city in Eastern China"

Dear Editor and Reviewers,

Thank you for allowing a resubmission of our manuscript, with an opportunity to address the reviewers’ comments. We are uploading (a) our point-by-point response to the comments (below) (response to reviewers), (b) a final manuscript with highlights (Revised Manuscript with Track Changes) (c) a clean final manuscript without highlights (Manuscript).

Best regards,

Authors: Cao Jianfei, Li Chunfang, Zhang Lixia, Wu Quanyuan, Lv Jianshu

**Reviewer#1, Concern # 1:** what do you mean by quantitavie analysis here. PCA and FA will provide quantitative shares of sources.

**Author response:** Thank for your comment. APCS/MLR evolved from PCA, and source contributions are obtained through carrying out the regressions between potentially toxic element contents and APCS. PMF uses experimental uncertainties in the data matrix and decomposes a data matrix into factor contributions and factor profiles under the non-negative constraint. These receptor models were able to calculate the concentration contribution of each pollutant source to each sample. PCA and FA, as a linear technique for dimensionality reduction, could transform many heavy metals into few principle component. Although they were able to get the loading and total variance, but cannot interpret the quantitative contributions of natural and human sources to heavy metals [1].

1	Lv JS. Multivariate receptor models and robust geostatistics to estimate source apportionment of heavy metals in soils. Environ Pollut. 2019; 244: 72-83. doi:10.1016/j.envpol.2018.09.147.

**Reviewer#1, Concern # 2:** receptor models have been widely used in aerosol scineces. You should mention this here and cite some literature (Chow, Pope, Dockery, ..,)

**Author response:** Thank for your advice. We have added an introduction to the application of the receptor model and cited valuable references.

14	Watson JG, Chow JC, Houck JE. PM2.5 chemical source profiles for vehicle exhaust, vegetative burning, geological material, and coal burning in northwestern Colorado during 1995. Chemosphere. 2001; 43 (8): 1141–1151.

**Reviewer#1, Concern # 3:** even earlier studies are available regarding geostatitstics in urban soils: Nathanail CP et al. 1998, Markus JA et al. 1996, Norra S et al. 2001, Norra S et al. 2006.

**Author response:** Thank for your advice. We have carefully reviewed and cited earlier literature.

19. Nathanail C P, Rosenbaum M S. Spatial management of geotechnical data for site selection [J]. Engineering Geology, 1998, 50(3-4): 347-356.
20. Markus J A, McBratney A B. An urban soil study: heavy metals in Glebe, Australia [J]. Soil Research, 1996, 34(3): 453-465.
21. Norra S, Lanka-Panditha M, Kramar U, et al. Mineralogical and geochemical patterns of urban surface soils, the example of Pforzheim, Germany [J]. Applied Geochemistry, 2006, 21(12): 2064-2081.

**Reviewer#1, Concern # 4:** Longkou is a ..., verb missing in this sentence。

**Author response:** We have revised this sentence.

**Reviewer#1, Concern # 5:** plain is not a material!

**Author response:** We have revised this sentence and figure.

**The main modifications were shown as follows (Lines 127-129):**

Parent material are composed of marine sediments in the western of study area, bordered by alluvium and moorstone in the southeastern region (**Fig 3**)

**Reviewer#1, Concern # 6:** reference for interviews? 28?

**Author response:** We have revised this sentence.

**Reviewer#1, Concern # 7:** reference?.

**Author response:** Thank for your comment. We added the corresponding literature

**Reviewer#1, Concern # 8:** why based on Landsat images?

**Author response:** Landsat Images is free data with high spatial resolution and strong timeliness.

**Reviewer#1, Concern # 9:** why 0.84 mm? This is quiet unusaual, since sand is smaller 2 mm and worldwide soil scientist agreed to analyse this fraction. Silt is smaller 63µm, but this also not fits to 0.84 mm.

**Author response:** We agree with your comment. To keep the soil smaller than 2mm, we used a 20-mesh (0.84mm) sieve to filter the soil. Unfortunately, we did not express this process clearly in the article. In the revised manuscript, we have updated the description of the experimental process.

**The main modifications were shown as follows (Lines 153-174):**

All subsamples were collected at a depth of 0-20 cm using a stainless-steel shovel. At each sampling site, an approximate 1kg of the soil sample were mixed thoroughly in a polyethylene bag. After air-drying, the collected soils samples were sieved to 2 mm, and ground to powder that could pass through a 0.149-mm mesh for physical-chemical analysis. The geographical locations of the sampling points were recorded by a GPS receiver, as shown in **Fig 1**.

In the laboratory, soil pH values were measured by a pH meter in a 1:2.5 soil–water suspension, and organic matter (SOM) contents were analyzed using oil bath-K_2_CrO_7_ titration [31]. H_2_SO_4_-HNO_3_-HF was used to digest the soil for analyzing the As, Cd, Cr, Cu, Hg, Ni, Pb and Zn contents. The Cr, Cu, Ni, Pb and Zn contents were analyzed using a flame atomic absorption spectrophotometer (240 AA Agilent, USA), Cd contents were determined using a graphite furnace atomic absorption spectrophotometer (AA-7000 Shimadzu, Japan), and As and Hg concentrations were determined with an atomic fluorescence spectrophotometer (AFS230E Haiguang Analytical Instrument Co., Beijing, China) following the digestion of H_2_SO_4_-HNO_3_-KMnO_4_ [31]. For details on the measurements, please refer to the related literature [24, 31]. A standard reference material, GSS-1 soil, obtained from the Center for National Standard Reference Material of China (http://www.biobw.com/), was used for quality control. The detection limits for each element are shown in the table 3. The recovery rate and standard reference material were examined under strict monitoring, and the chemical analysis process followed the standard for geochemical evaluation of land quality (DZ/T0295—2016) in China. The limit of recovery was 94%~106%.

**Reviewer#1, Concern # 10:** You milled 1 kg?????

**Author response:** In the revised manuscript, we have updated the description of the experimental process.

**The main modifications were shown as follows (Lines 153-174):**

All subsamples were collected at a depth of 0-20 cm using a stainless-steel shovel. At each sampling site, an approximate 1kg of the soil sample were mixed thoroughly in a polyethylene bag. After air-drying, the collected soils samples were sieved to 2 mm, and ground to powder that could pass through a 0.149-mm mesh for physical-chemical analysis. The geographical locations of the sampling points were recorded by a GPS receiver, as shown in **Fig 1**.

In the laboratory, soil pH values were measured by a pH meter in a 1:2.5 soil–water suspension, and organic matter (SOM) contents were analyzed using oil bath-K_2_CrO_7_ titration (Lu, 2000).

**Reviewer#1, Concern # 11:** it is already air dried.

**Author response:** We have revised it.

**Reviewer#1, Concern # 12:** Did you analyse soil pH at milled soil sample? This is problematic, since you know have more and perhaps other surfaces and this pH must not reflect the soil's pH. pH is measured at soil fraction < 2 mm.

**Author response:** In the revised manuscript, we have updated the description of the experimental process.

**The main modifications were shown as follows (Lines 153-174):**

All subsamples were collected at a depth of 0-20 cm using a stainless-steel shovel. At each sampling site, an approximate 1kg of the soil sample were mixed thoroughly in a polyethylene bag. After air-drying, the collected soils samples were sieved to 2 mm, and ground to powder that could pass through a 0.149-mm mesh for physical-chemical analysis. The geographical locations of the sampling points were recorded by a GPS receiver, as shown in Fig 1.

**Reviewer#1, Concern # 13:** which purity grades, which amounts, detection limits, reproducibility of standard material concnetrations?

**Author response:** Thank for your comment. A standard reference material, GSS-1 soil, obtained from the Center for National Standard Reference Material of China (http://www.biobw.com/), was used for quality control. The chemical analysis process followed the standard for geochemical evaluation of land quality (DZ/T0295—2016) in China. The detection limits of our method are shown in Table 1. As far As we know, chemical analysis of soil element (As, Cd, Cr, Cu, Hg, Ni, Pb and Zn) is a normal experiment. In order to ensure the accuracy of the experiment, relevant standard materials have been are developed locally. The relevant standard specification represents the experimental reproducibility. These standard specifications represent experimental reproducibility. Therefore, it is not necessary for us to have too much knowledge about popular science in this article, which identify and apportion sources of soil potentially toxic elements.

**Table 1** **Detection limits of the method (****ng mL^-1^**)

| Element |  | As | Cd | Cr | Cu | Hg | Ni | Pb | Zn |
| --- | --- | --- | --- | --- | --- | --- | --- | --- | --- |
| Detection limits |  | 0.3 | 0.01 | 0.5 | 0.1 | 0.001 | 0.3 | 0.1 | 0.2 |

**In the revised manuscript, we have added a description of the necessary experimental procedures. The main modifications were shown as follows (Lines 169-174):**

A standard reference material obtained from the Center for National Standard Reference Material of China (http://www.biobw.com/), was used for quality control. The recovery rate and standard reference material were examined under strict monitoring, and the chemical analysis process followed the standard for geochemical evaluation of land quality (DZ/T0295—2016) in China.

**Reviewer#1, Concern # 14:** It is not enough just to refer some other literature regarding to verify the exactness of your analysis. You must have shown at least the reproducibility within your study by your own with your standard material. You also have to mention which standard material was used. What does it mean, limit of recovery? Did you eliminate other results? Provide a table with the results for the standard material.

**Author response:** The recovery rate was used to confirm the accuracy of this experiment. The standard recovery results of each element are shown in Table 4. In this paper, we only use the recovery rate to prove that the results of this experiment are reliable and the experimental process is mature, which does not need too much description.

**Table 2 Recovery of the method (ng mL^-1^**)

| Element |  | As | Cd | Cr | Cu | Hg | Ni | Pb | Zn |
| --- | --- | --- | --- | --- | --- | --- | --- | --- | --- |
| Recommended value |  | 3.0 | 0.21 | 87 | 37 | 0.092 | 41 | 38 | 94 |
| Addition |  | 10 | 0.3 | 100 | 50 | 0.1 | 50 | 50 | 100 |
| Measured value |  | 12.8 | 0.52 | 191 | 90 | 0.190 | 88 | 88 | 196 |
| Recovery/% |  | 98.5 | 103.3 | 104 | 106.0 | 98.0 | 94.0 | 94.0 | 102 |

**The main modifications were shown as follows (Lines 153-174).**

The limit of recovery was 94%~106%.

**Reviewer#1, Concern # 15:** Add a formular for APCS calculation.

**Author response:** In the revised manuscript, we have updated the description of APCS. Factor fractions of these normalized heavy metal concentrations can be obtained by using PCA. APCS can be obtained from the factor score of each sample minus the factor score of the 0 concentration sample. The corresponding regression coefficient can be obtained by using multiple linear regression of APCS with metal concentration data

**The main modifications were shown as follows (Lines 196-197):**

The APCS for the factors are estimated by subtracting the factor scores of Z_0_ from the factor scores of true samples

**Reviewer#1, Concern # 16:** factor profile? isn't it the standard deviation?.

**Author response:** In the revised manuscript, we have updated the description of the formula.

**The main modifications were shown as follows (Lines 212-213):**

where σ_ij_ is the uncertainty in the ith chemical element for sample j

**Reviewer#1, Concern # 17:** C_0_/C_0_+C equals to C!.

**Author response:**

Fig.1 Diagram of ideal variation

An idealized variation curve is shown in Fig.1. Where, C_0_ is Nugget variance, C_0_+C is the Sill, which is a basically stable constant obtained after the semi-variance increases to a certain degree with the distance. C is the variance of the structure, which the difference between the Nugget value and the Nugget coefficient. The ratio of C_0_/C_0_+C is an important indicator of spatial heterogeneity of a regionalized variable, also known as Nugget effect.

**Reviewer#1, Concern # 18:** a table with all single data in the supplementaries would be helpful for reproduction of the calcualtions

**Author response:** In the supporting information, we have provided a table with all single data.

**Reviewer#1, Concern # 19:** what do you mean here by species?

**Author response:** In the revised manuscript, we have revised it.

**Reviewer#1, Concern # 20:** present - or? They still do.

**Author response:** Thanks for your kindly suggestion. We have revised it.

**Reviewer#1, Concern # 21:** according to table 1 it is only 27.1%. Also the other variances in the text do not fit to the table 1

**Author response:** Thanks for your kindly suggestion. We have revised it.

**The main modifications were shown as follows (Lines 196-197):**

The first factor (F1) accounted for 27.15% of the total variance and showed strongly positive loadings of Cr and Ni and a moderate loading of As. F2 explained approximately 18.94 % of the total variation and had a highly positive loading of Cu and a moderate loading of Zn. F3 explained 17.64% of the total variance and had a highly positive loading of Pb and a moderate loading of Cd. F4 accounted for 15.88% of the total variance and a highly positive loading of Hg.

**Reviewer#1, Concern # 22:** R² of what? **(Line 278)**

**Author response:** The Ratio (predicted/observed) values was also used to evaluate the results of these receptor models.

**Reviewer#1, Concern # 23:** Did you carry out cross validations?

**Author response:** Yes, these results have been cross-validated using ArcGIS 10.1 (ESRI Inc., USA).

**Reviewer#1, Concern # 24:** This subchapter is more or less a repitition of the results - not really necessary here.

**Author response:** Thanks for your kindly suggestion. To refine the article, we removed it.

**Reviewer#1, Concern # 25:** Citing Alloway here is misleading in this context since the statement cannot be generalized. There are cases in which Cr and Ni derive from intensive anthropogenic activities and do not reflect primarily the geogenic background.

**Author response:** Thanks for your kindly suggestion. In the revised manuscript, we corrected the inaccurate expression.

**Reviewer#1, Concern # 26:** You just picked studies where Cr and Ni are of geogenic origin. There are many cases when elevated concentrations are of anthropogenic conc. However, your Cr and Ni are quiet low, so it is clear that in your case the geogenic context is more important than the human context.

**Author response:** Thanks for your kindly suggestion. In the revised manuscript, we corrected the inaccurate expression.

**Reviewer#1, Concern # 27:** You explain the sources by citing other studies. That is ok, but I miss the classical first studies and compilations of those source studies as from Wedepohl, Nriagu or Merian from the 70th or 80th.

**Author response:** Thanks for your kindly suggestion. We have added references to classic studies.

49	Nriagu JO. Global metal pollution: poisoning the biosphere? Environment: Science and Policy for Sustainable Development. 1990; 32(7): 7-33.

50	Wedepohl KH. The composition of the continental crust. Geochim. Cosmochim. Acta. 1995; 7:1217–1232

**Reviewer#1, Concern # 28:** You should not speculate in a scientific study

**Author response:** We have revised it.

**Reviewer#1, Concern # 29:** 101%???? not 100%?

**Author response:** In total, the solutions of the APCS/MLR results explained approximately 101% of all the sources. The reason for this result is that APCS/MLR had some negative and unidentified contributions.

**Reviewer#1, Concern # 30:** why not showing this result in a pie diagram?

**Author response:** Thanks for your kindly suggestion. In the manuscript, the source contributions of potentially toxic elements are presented in Table 3. If we add pie diagrams, there will be repeated descriptions of the content in the article.

**Reviewer#1, Concern # 31:** The study is quiet local. I propose to add here results from other cities and urbanized regions to integrate these results into the international state of research and to compare with the pollution levels of other cities.

**Author response:** Thanks for your kindly suggestion. In the revised manuscript, we analyzed the characteristics of heavy metals sources in China and the level of the study area.

**The main modifications were shown as follows (Lines 441-454):**

“

At the Chinese scale, Hg dramatically declined due to strict control of atmospheric Hg emission in China since 2010 [66]. Moreover, inputs of all the heavy metals from fertilizers decreased, because of the stricter fertilizer management and modernized fertilizer production technologies [66]. Heavy metals are more likely to be enriched from fertilizers and sewage irrigation sources in North China, where water is scarce, than in the South China [67-68]. In provinces with high GDP (Guangdong, Jiangsu, Henan, and Shandong provinces), industrial and traffic activities sources contributed more heavy metals in soil, mainly Pb and Cd [69]. In general, the source of soil potentially toxic element in the study area is similar to that in China as a whole. The law of the People’s Republic of China on the prevention and control of soil pollution was came into effect on January 1, 2019, it constitute a new comprehensive control system of soil pollution. In the future, local governments can develop more effective soil pollution control strategies based on studies of the quantitative sources of potentially toxic element.

66	Ni R, Ma Y. Current inventory and changes of the input/output balance of trace elements in farmland in China nationwide. PLoS One. 2018;13 (6): e0199460

67	Hao P, Chen YL, Weng LP, Ma J, Ma YL, Li YG, Islam MS. Comparisons of heavy metal input inventory in agricultural soils in North and South China: a review. Science of the Total Environment. 2019; 660: 776-786.

68	Zhang Q, Wang C. Natural and Human Factors Affect the Distribution of Soil Heavy Metal Pollution: a Review. Water Air Soil Pollut, 2020, 231(7): 1-13.

69	Hu BF, Shao S, Ni H, Zhao Y, Min XX, She SF, Chen SC, Huang MX, Zhou LQ, Li Y, Shi Z. Current status, spatial features, health risks, and potential driving factors of soil heavy metal pollution in China at province level. Environ Pollut. 2020; 266:114961.

Thank you very much for your valuable comments. We have polished the manuscript and the revised manuscript has been submitted. We look forward to your positive response.

Best regards,

The authors
